# Purifying circRNA by ultrafiltration with membranes having well-defined pores

**DOI:** 10.1016/j.seppur.2026.137031

**Published:** 2026-01-28

**Authors:** Karen Guillen-Cuevas, Caroline C. Hansen, Deepraj Sarmah, Marc R. Birtwistle, Scott M. Husson

**Affiliations:** Department of Chemical and Biomolecular Engineering, Clemson University, 127 Earle Hall, 206 S. Palmetto Blvd., Clemson, SC 29634, USA

**Keywords:** circRNA therapeutics, circRNA manufacturing, RNA continuous diafiltration, RNA gene expression

## Abstract

Circular RNA (circRNA) is a promising therapeutic modality owing to its enhanced stability conferred by exonuclease resistance. However, large-scale production remains limited by inefficient purification methods, as the standard approach using size-exclusion chromatography suffers from peak overlap and low yield. Here, we present an ultrafiltration strategy for purifying circRNA using polycarbonate track-etched membranes with precisely controlled pore diameters. Membrane pore sizes were fine-tuned by gold plating to separate circular and linear RNA conformers. Sieving coefficients of purified RNA conformers were measured to establish operating conditions, and continuous diafiltration experiments were conducted within the range of flux values predicted to achieve both high purity and yield. This approach resulted in 94% purity and 59% yield of circRNA from *in vitro* transcription mixtures after six diavolumes. Transfection studies in HEK293T cells confirmed that diafiltered circRNA exhibited gene expression levels comparable to enzymatically purified circRNA, albeit with modestly reduced transfection efficiency. These results establish membrane-based ultrafiltration with tailored pore sizes as a scalable, non-chromatographic platform for purifying circRNA and provide a framework for further development toward therapeutic circRNA manufacturing.

## 1. Introduction

Circular RNA (circRNA) is emerging as a new therapeutic modality, offering exceptional stability and resistance to exonuclease degradation, which is conferred by its covalently closed structure lacking 5′ and 3′ ends. This stability enables sustained translation in target cells without genomic integration [1], reducing the need for repeated dosing and supporting long-lasting therapeutic effects. Such precise and durable gene therapies hold promise for revolutionizing the treatment of many genetic and acquired diseases, ushering in a new era of safer and more effective interventions [2].

CircRNA is synthesized using *in vitro* transcription (IVT) and a self-splicing or enzymatic circularization reaction. The resulting crude transcription mixture contains a complex blend of species, including various RNA molecules: uncircularized RNA precursors, intact circRNA, circular RNA concatamers (multimers of a circRNA sequence), and nicked RNA, which is RNA that was circularized but damaged during the process and rendered nonfunctional [3]. Purifying intact circRNA from this mixture is essential for therapeutic use; however, the state-of-the-art method, size-exclusion high-performance liquid chromatography (SE-HPLC), suffers from severe peak overlap, resulting in low yield and making it unsuitable for large-scale manufacturing [4].

We previously demonstrated that ultrafiltration can be used to purify circRNA [5]. However, when using polyethersulfone membranes, the pore size distribution allowed a small fraction of circRNA to pass through, resulting in a non-zero sieving coefficient within the studied pressure range and reducing the overall yield. Polycarbonate track-etched (PCTE) membranes have a significantly narrower pore size distribution; however, commercially available pore sizes were not suitable for circRNA purification. Knowing that gold plating can be used to tailor pore diameter precisely [6], we hypothesized that tuning the pore size *via* gold plating could produce a membrane well-suited for the high-purity, high-yield separation of circular and linear RNA. Purified RNA molecules were used to measure the individual sieving coefficients and identify conditions leading to effective purification. These conditions were tested to purify circRNA from IVT reaction solutions and compare the transfection efficiency and gene expression of the UF-purified circRNA with that treated enzymatically to remove linear RNA contaminants.

## 2. Materials and methods

### 2.1. Plasmid and DNA template

The bacterial plasmid used to transcribe circRNA encoding mNeon-Green (circRNA-synIRES-R25-mNeonGreen) was a gift from Howard Chang (Addgene Plasmid #188115, Watertown, MA, USA) [3]. The plasmid was streak-plated onto an agar plate (Fisher Bioreagents, Suwanee, GA, USA) containing 50 μg/mL kanamycin (Apexbio Technology LLC, Houston, TX, USA) and incubated for 18 h at 37 °C. Individual colonies were inoculated into LB media (Fisher Bioreagents) supplemented with 50 μg/mL kanamycin (Apexbio) and grown for 18 h at 37 °C. Plasmid DNA was extracted from the bacterial culture using the ZymoPURE Plasmid Miniprep Kit (Zymo Research, Fisher Scientific, Suwanee, GA, USA). The plasmid was linearized and amplified by polymerase chain reaction (PCR) using the Q5 High-Fidelity PCR Kit (New England Biolabs, Ipswich, MA, USA) according to the manufacturer's instructions, with an annealing temperature of 70 °C. Primers were designed by Howard Chang and synthesized by Integrated DNA Technologies (Coralville, IA, USA).

### 2.2. CircRNA synthesis and purification

CircRNA was synthesized using the HiScribe T7 High Yield RNA Synthesis Kit (New England Biolabs, Ipswich, MA, USA) and the circRNA-synIRES-R25-mNeonGreen DNA template. One microgram of DNA template was used in a 20 μL reaction, following the manufacturer's instructions. The resulting crude IVT mixture was treated with DNase I, as previously described [5], to degrade the DNA template. The treated IVT mixture was cleaned using the Monarch Spin RNA Cleanup Kit (500 μg) (New England Biolabs) to eliminate the enzyme and linear RNA fragments and then stored at −80 °C until use.

### 2.3. Probe circularization

For probing circularization, crude IVT mixtures were treated with RNase R (New England Biolabs), a highly processive 3′ to 5′ exoribonuclease that selectively digests linear RNA. One microliter of RNase R (20 units) and 2 μL of 10× RNase R Reaction Buffer were used for 20 μg of crude IVT mixture. The samples were incubated at 37 °C for 30 min. The digested circRNA was cleaned using the Monarch Spin RNA Cleanup Kit (500 μg) (New England Biolabs) to remove enzyme and linear RNA fragments.

RNase R-digested IVT mixtures were analyzed on *E*-Gel 2% EX Agarose Gels (Invitrogen, Thermo Fisher Scientific, Waltham, MA, USA) to identify the band corresponding to circRNA. For each sample, ~200 ng of RNA and 1.5 μL of ssRNA ladder (New England Biolabs) were diluted to a total volume of 10 μL, followed by the addition of 10 μL of 2× RNA loading dye (New England Biolabs). Unused wells were loaded with 20 μL of diethylpyrocarbonate (DEPC)-treated water. Gels were run for 15 min in an *E*-Gel Power Snap Electrophoresis Device (Thermo Fisher Scientific) using the “E-Gel EX 1–2%” setting, then placed on ice for 5 min^7^. Images were captured using a Bio-Rad ChemiDoc XRS+ imaging system (Bio-Rad Laboratories, Hercules, CA, USA) with Image Lab 6.1 software and the “SYBR-GOLD” setting.

### 2.4. Obtaining purified RNA conformers from crude IVT mixtures

To obtain purified fractions of circular and linear RNA conformers, ~4 μg of IVT reaction mixture was diluted to a final volume of 100 μL and mixed with 100 μL of 2× RNA loading dye (New England Biolabs). Two microliters of ssRNA ladder (New England Biolabs) were diluted to a total volume of 10 μL and mixed with 10 μL of 2× RNA loading dye. *E*-Gel 2% EX Agarose Gels were loaded with 20 μL of ladder-dye solution in the first well and 20 μL of RNA-dye solution in each of the remaining 10 wells. Gels were run for 15 min in an *E*-Gel Power Snap Electrophoresis Device using the “E-Gel EX 1–2%” setting and then placed on ice for 5 min. Once the bands were clearly visible under blue light, the cassette was opened using a sterile flathead screwdriver and returned to the device. Wearing orange safety glasses, the desired bands were excised with a sterile razor and transferred into pre-weighed 1.5 mL tubes. RNA was extracted using the Zymoclean Gel RNA Recovery Kit (Fisher Scientific) according to the manufacturer's instructions, with the following modification: gel melting was performed by incubating the samples at 50 °C for 30 min with mixing every 10 min. In the final step, 15 μL of DNase/RNase-free water was added, and the samples were incubated for 10 min at ambient temperature (~24 °C) and then centrifuged at 16000*g* for 1 min (Model: 75002411, Thermo Fisher Scientific).

### 2.5. Membrane selection

Polycarbonate track-etched (PCTE) membranes were sourced from Sterlitech (Auburn, WA, USA). Membranes with pore diameters of 10, 30, and 50 nm were studied.

### 2.6. Gold plating PCTE membranes

PCTE membranes were immersed for 3 min in a solution of 0.026 M SnCl_2_ (95% Strem Chemicals, Newburyport, MA, USA) and 0.07 M trifluoroacetic acid (99%, Sigma Aldrich, Burlington, MA, USA) prepared in a 50:50 (*v*/v) methanol/water solution. Membranes were rinsed three times with methanol (Sigma-Aldrich), with a Whatman filter paper (Cytiva, Marlborough, MA, USA) placed over the membrane during the final rinse to prevent curling. After methanol removal, 10 mL of aqueous ammoniacal AgNO_3_ (0.029 M; Thermo Fisher Scientific, Waltham, MA, USA) was added, and the membranes were incubated for 5 min. Membranes were then immersed in methanol and placed on a Fisher brand shaker (Pittsburgh, PA, USA) for 5 min, followed by two additional methanol rinses, using Whatman filter paper during the final rinse.

Gold plating was performed by immersing the membranes in an ice-cooled gold plating bath. The plating solution was prepared using 0.25 mL of a gold stock solution described in the patent [8], in which the elemental gold concentration was 21.2 g/L in the form of Na_3_[Au(SO_3_)_2_]. This aliquot was diluted to a final volume of 10 mL with water and reagents to yield a gold plating bath containing 0.127 M Na_2_SO_3_ (Sigma-Aldrich), 0.625 M formaldehyde (37%, Thermo Scientific), and 0.025 M NaHCO_3_ (>99.5%, Sigma-Aldrich). The pH was adjusted to 10 using 1 M sulfuric acid (95–98%, Sigma-Aldrich), and all plating reactions were carried out in an ice bath. Membranes were plated for 45, 50, or 60 min, rinsed with water, and subsequently immersed in a 15% (*v*/v) nitric acid (70% vol, Sigma-Aldrich) solution for 16 h. After a final rinse, membranes were stored in 10% (v/v) methanol in water until use.

### 2.7. Measuring membrane hydraulic permeability

The membrane was removed from the water/methanol storage solution and placed on the base of a 50 mL Amicon Stirred Cell (Millipore Sigma, Burlington, MA, USA) with 3MM CHR grade Whatman chromatography paper (Cytiva) underneath. An o-ring was placed above the membrane to seal the edges. The membrane was conditioned by filtering 50 mL of water at 414 kPa, after which the stirred cell was refilled with DEPC-treated water. The stirring speed for all measurements was 200 rpm.

Flux was measured at three transmembrane pressures (414, 276, and 138 kPa), with three replicate measurements taken at each pressure, starting from the highest pressure. For each measurement, the initial mass of the permeate collector was recorded. Water was collected for approximately 30 s. The change in mass was divided by the collection time and the density of water to determine the volumetric flow rate. Flux was calculated by dividing the volumetric flow rate by the exposed membrane surface area (13.4 cm^2^). The pressure sequence was repeated in decreasing and increasing order. Hydraulic permeability was determined from the slope of the flux-pressure plot.

### 2.8. Measuring RNA sieving coefficients

A 10 mL tris-ethylenediaminetetraacetic acid (TE) buffer solution was prepared by diluting 500 μL of 20× TE buffer (Thermo Fisher Scientific) in 9.5 mL of DEPC-treated water. RNA was added to achieve a final concentration of ~30 ng/mL, and the solution was transferred to a 50 mL Amicon Stirred Cell (Millipore Sigma) with the membrane in place. A 100 μL sample was collected to determine the initial concentration. Afterward, the cell was pressurized at 414 kPa, and the stirring speed was set to 200 rpm. The permeate valve was opened, and 2 mL of permeate was collected, followed by a 100–200 μL sample for concentration analysis. The pressure was released, and a 100 μL retentate sample was collected. The procedure was repeated for all pressure points.

RNA concentration was measured using the Quant-iT RiboGreen RNA Reagent and Kit (Thermo Fisher Scientific). For each 100 μL RNA sample, 100 μL of diluted dye was added, and the 200 μL mixture was transferred to a 96-well black plate with a clear bottom. The plate was incubated for 5 min in the dark, covered with aluminum foil. Fluorescence was measured using a Synergy H1 microplate reader (excitation 485 nm, emission 530 nm) (Agilent, Santa Clara, CA, USA). Concentrations were determined from a standard curve. The sieving coefficient (S) was calculated as the quotient of the permeate concentration and the average retentate concentration, calculated from samples taken immediately before and after permeate collection.

### 2.9. Continuous diafiltration of IVT reaction mixtures.

A 50 mL TE buffer solution for the IVT RNA reaction mixture and a 300 mL TE buffer solution for the reservoir were prepared using Invitrogen 20× RNase-free TE Buffer (Thermo Fisher Scientific). The IVT reaction mixture was added to the 50 mL of TE buffer solution to achieve a concentration of ~60 ng/mL, and the solution was transferred to the stirred cell holding the membrane. A 100 μL sample was collected to measure the initial concentration.

The valves connecting the stirred cell to the reservoir were set to batch mode, and the compressed air valve was opened. The stirred cell was placed into an aluminum bead bath maintained at 4 °C. Once the stirred cell and the reservoir were pressurized, the stirring speed was set to 200 rpm, and the 3-way valve was switched from batch to continuous mode. At that point, the feed solution began flowing from the reservoir into the stirred cell. Every 30 min, the flux was measured by recording the mass of the permeate collector over time. The pressure was adjusted to maintain the flux near the target value, with minor deviations resulting from the manual control process.

Diafiltration continued until all TE buffer in the reservoir was consumed or the target number of diavolumes was reached. The number of diavolumes is a unit of measure for the total buffer volume added during a diafiltration process. It is calculated by dividing the total volume of diafiltration buffer by the initial volume of the feed solution in the cell. The compressed air valve was closed, and the system was depressurized by opening the relief valve. A 100 μL sample was then taken to measure the final retentate concentration.

For concentrating the retentate, the stirred cell was removed from the aluminum bead bath and repressurized in batch mode to 350 kPa. Filtration continued until the retentate volume was reduced to <10 mL. A 100 μL sample was collected for concentration analysis, and the remaining retentate was aliquoted into 400 μL portions for ethanol precipitation.

### 2.10. Purity determination

Initially, purity was calculated using *E*-Gel^®^ 2% EX Agarose Gels by performing densitometric integration of bands. In cases where the concentration of linear RNA was too low to be reliably quantified by gel analysis, purity was determined using a functional degradation approach: total RNA concentration was measured using the Quant-iT RiboGreen assay, and purity was calculated from the fraction of RNase R-resistant RNA. This approach provides a means of determining circRNA enrichment but does not constitute a direct structural identification of RNA species.

To provide orthogonal, more specific quantification of circRNA, RT-qPCR with junction-spanning primers was performed on selected diafiltration samples. CircRNA was reverse transcribed into cDNA using the Invitrogen SuperScript^™^ IV First-Strand Synthesis System, and qPCR was performed using iTaq^™^ Universal Supermix. CircRNA concentration was determined from standard curves, and sample purity was calculated as the ratio of circRNA concentration to total RNA concentration measured using the Quant-iT RiboGreen assay.

### 2.11. Ethanol precipitation of RNA

One milliliter of absolute ethanol (99.5%, Thermo Fisher Scientific), 40 μL of 3 M sodium acetate (pH 5.2, Sigma Aldrich), and 4 μL of RNA glycogen (Thermo Fisher Scientific) were added to each 400 μL aliquot of diafiltration product. Samples were incubated at −20 °C for 16 h and centrifuged at 4 °C for 30 min at 16000 g. The supernatant was removed, and the pellet was washed with 700 μL of prechilled 70% (*v*/v) aqueous ethanol at 4 °C. Washes were followed by centrifugation at 4 °C for 15 min at 16000 g. The supernatant was discarded, and the pellet was resuspended in 5 μL of TE buffer.

### 2.12. Transfection of diafiltrated and RNase R-digested circRNA into HEK293T cells

For the 96-well culture plate (Falcon, Colorado Springs, CO, USA), 2 × 10^4^ HEK293T cells were plated per well 24 h before transfection. For the 12-well plate (Falcon), 4 × 10^6^ cells were diluted to a final volume of 4 mL using Dulbecco's Modified Eagle Medium (DMEM) (Gibco, Thermo Fisher Scientific) supplemented with 10% Fetal bovine serum (FBS) (Thermo Fisher Scientific) and seeded at 1 mL per well in a 12-well culture plate 24 h before transfection. Cells at 70–90% confluency were used for transfection. Lipofectamine 2000 reagent (24 μL, Invitrogen, Thermo Fisher Scientific) was diluted to a final volume of 300 μL in Opti-MEM medium (Thermo Fisher Scientific) and incubated at ambient temperature (~24 °C) for 5 min. Separately, 500 ng of diafiltered circRNA and 500 ng of RNase R-digested circRNA were each diluted to a final volume of 100 μL in Opti-MEM medium. The following conditions were prepared: Well 1 (untreated control) consisted of 200 μL of Opti-MEM medium only. Well 2 (Lipofectamine control) consisted of 100 μL Lipofectamine 2000 solution mixed with 100 μL of Opti-MEM medium. Well 3 (diafiltered circRNA) consisted of 100 μL Lipofectamine 2000 solution mixed with 100 μL of diafiltrated circRNA solution. Well 4 (RNase R-digested circRNA) consisted of 100 μL Lipofectamine 2000 solution mixed with 100 μL of RNase R-digested circRNA solution. The culture plate was incubated at 37 °C with 5% CO_2_ for 20 h.

### 2.13. Flow cytometry

Cellular fluorescence was first verified using a Revolve Fluorescence Microscope (ECHO, San Diego, CA, USA) before initiating flow cytometry. The transfection efficiency was estimated using Cell Profiler to analyze the microscope images. For flow cytometry, culture medium was removed, and the cells were washed with 0.5 mL phosphate-buffered saline (PBS) (Fisher Bioreagents, Fisher Scientific) at 37 °C per well. Cells were detached using 0.5 mL TrypLE Enzyme Express (1×, Thermo Fisher Scientific) per well. After 2 min, 0.5 mL of DMEM 10% FBS medium at 37 °C was added to each well to neutralize the TrypLE, and the cell suspension was collected into a 1.5 mL tube on ice.

The tubes were centrifuged at 300 *g* for 5 min; the supernatant was discarded, and the pellets were resuspended in 500 μL of PBS containing 1% (*w*/*v*) FBS. The flow cytometer (Cytek Aurora, San Diego, CA, USA) was powered on one hour before use. Instrument performance and setup were verified using SpectroFlo QC beads (Lot 2005) according to the manufacturer's instructions. Samples were run in the following order: (1) Untransfected HEK293T cells used to adjust the side scatter (SSC) and forward scatter (FSC) parameters and gate for singlets. (2) HEK293T cells treated with Lipofectamine 2000 only, which was used as a negative control. (3) HEK293T cells transfected with diafiltered circRNA or RNase R-digested circRNA. The samples were analyzed using the green fluorescent protein (GFP) setting (488 nm excitation, 530 nm emission).

## 3. Results

### 3.1. CircRNA synthesis, purification, and RNase R-based characterization

Gradient PCR was performed to determine that 70 °C was the ideal annealing temperature for the DNA template. Following the IVT reaction, a sample of the IVT reaction mixture was treated with RNase R, an exonuclease that degrades linear RNA but not circRNA, and analyzed by gel electrophoresis alongside untreated samples.

Although circular and linear RNA derived from the same transcript have identical nucleotide lengths, they exhibit distinct electrophoretic migration behavior arising from differences in molecular topology. As reported by Abe et al. [7], circular and linear RNAs can display distinct characteristic migration patterns under agarose gel electrophoresis.

**Fig. 1.**
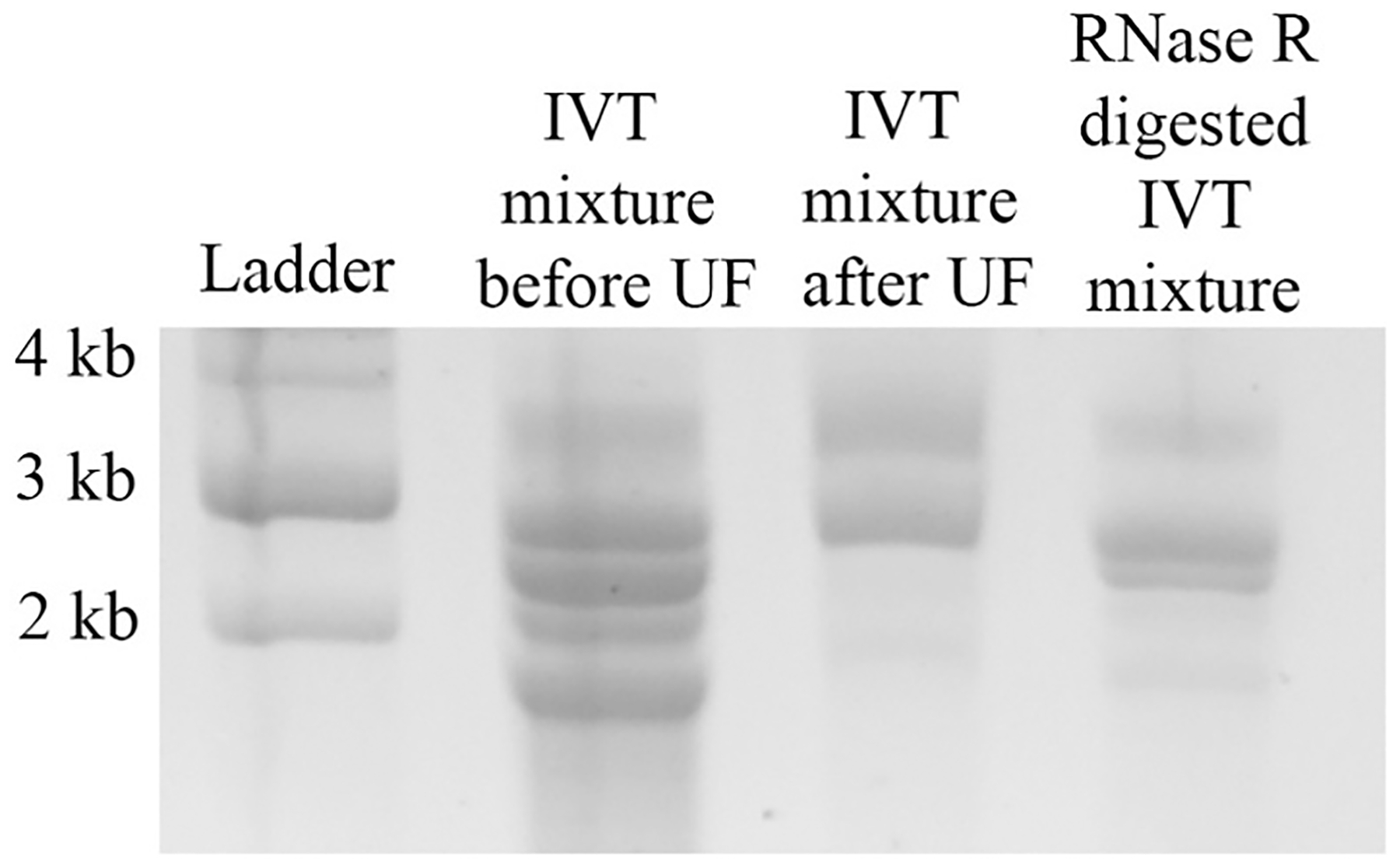
Agarose gel electrophoresis of IVT products before and after ultrafiltration and RNase R digestion. Lane 1 after the ladder: IVT mixture prior to ultrafiltration. Lane 2: IVT mixture in the retentate following ultrafiltration. Lane 3: RNase R-treated IVT mixture used for functional identification of RNase R-resistant (circRNA) species. Differences in migration reflect topology-dependent behavior rather than nucleotide length.

Comparison of untreated and RNase R-treated IVT mixtures (Fig. 1) enabled functional assignment of RNA species. RNase R-resistant bands were interpreted as circRNA species, whereas bands susceptible to RNase R digestion were attributed to linear RNA. Two RNase R-resistant bands were observed; the more slowly migrating species is assumed to represent a circular concatemer based on its resistance to RNase R digestion and its reduced electrophoretic mobility relative to the primary circRNA species. This interpretation is based on functional behavior rather than definitive structural validation.

### 3.2. Membrane selection

**Fig. 2.**
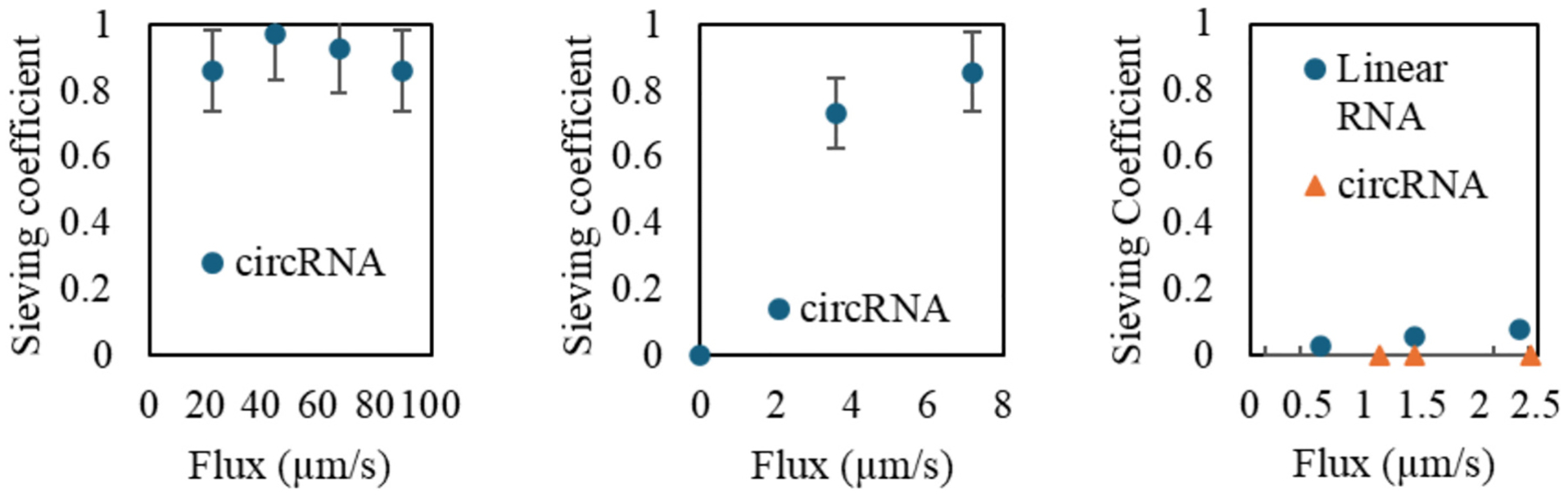
Sieving coefficients of purified fractions at different membrane pore diameters. Left) 50 nm. Middle) 30 nm. Right) 10 nm.

Purified fractions of circRNA and linear RNA from gel extraction were used to determine a suitable membrane pore diameter (Fig. 2). For the 50 nm membrane, the circRNA sieving coefficient was high (0.85–0.98) across the entire flux range, indicating that the circRNA predominantly transmitted through the membrane with minimal retention. The 30 nm membrane exhibited strong flux dependence: the circRNA sieving coefficient increased sharply from approximately 0.15 to 0.9 over a narrow flux range, demonstrating partial transmission and a clear transition region that ultimately resulted in near-complete transmission at higher flux. Linear RNA was not tested for the 30 nm membrane because numerous prior experiments have observed that the sieving coefficient of linear RNA is higher than that of circRNA with the same nucleotide length. Consequently, if transmission of circRNA across the membrane is observed, it can be inferred that linear RNA would also be transmitted. For the 10 nm membrane, the sieving coefficients of circRNA were zero, indicating complete retention, while those for linear RNA approached zero, indicating near-complete retention. These results suggest that the ideal pore diameter for separation is between 10 and 30 nm. The 30 nm membrane is too permissive for high-purity purification. In contrast, the low sieving coefficients for linear RNA at 10 nm would require impractically large buffer volumes to achieve a target purity above 90%. Therefore, we pursued fine-tuning of pore sizes to approximately 25 nm using gold plating techniques to achieve high-purity separation under practical operating conditions.

### 3.3. Hydraulic permeability of gold-plated PCTE membranes

We evaluated the effects of gold plating solution concentrations and plating time on membrane permeability and pore diameter. Table 1 summarizes the results obtained for membranes plated under different conditions. Pore diameters were estimated from the change in total flow rate (Q_total_) using the Hagen-Poiseuille Equation. Δp is the transmembrane pressure, μ is the dynamic viscosity, and L is the length of the pore. The total flow rate is given by Eq. (1), where N is the total number of pores. After gold plating, the number of pores remains the same, N_1_ = N_2_. Thus, for constant pressure filtration, Eq. (2) relates the pore diameters before and after plating to the flow rates.


(1) $$Q_{\text{total}}=\frac{N\pi \Delta pd^{4}}{\mu L}$$

(2) $$\frac{Q_{\text{total}\;1}}{d_{1}^{4}}=\frac{Q_{\text{total}\;2}}{d_{2}^{4}}$$

### 3.4. Sieving coefficient of RNA conformers in IVT reaction mixtures

**Fig. 3.**
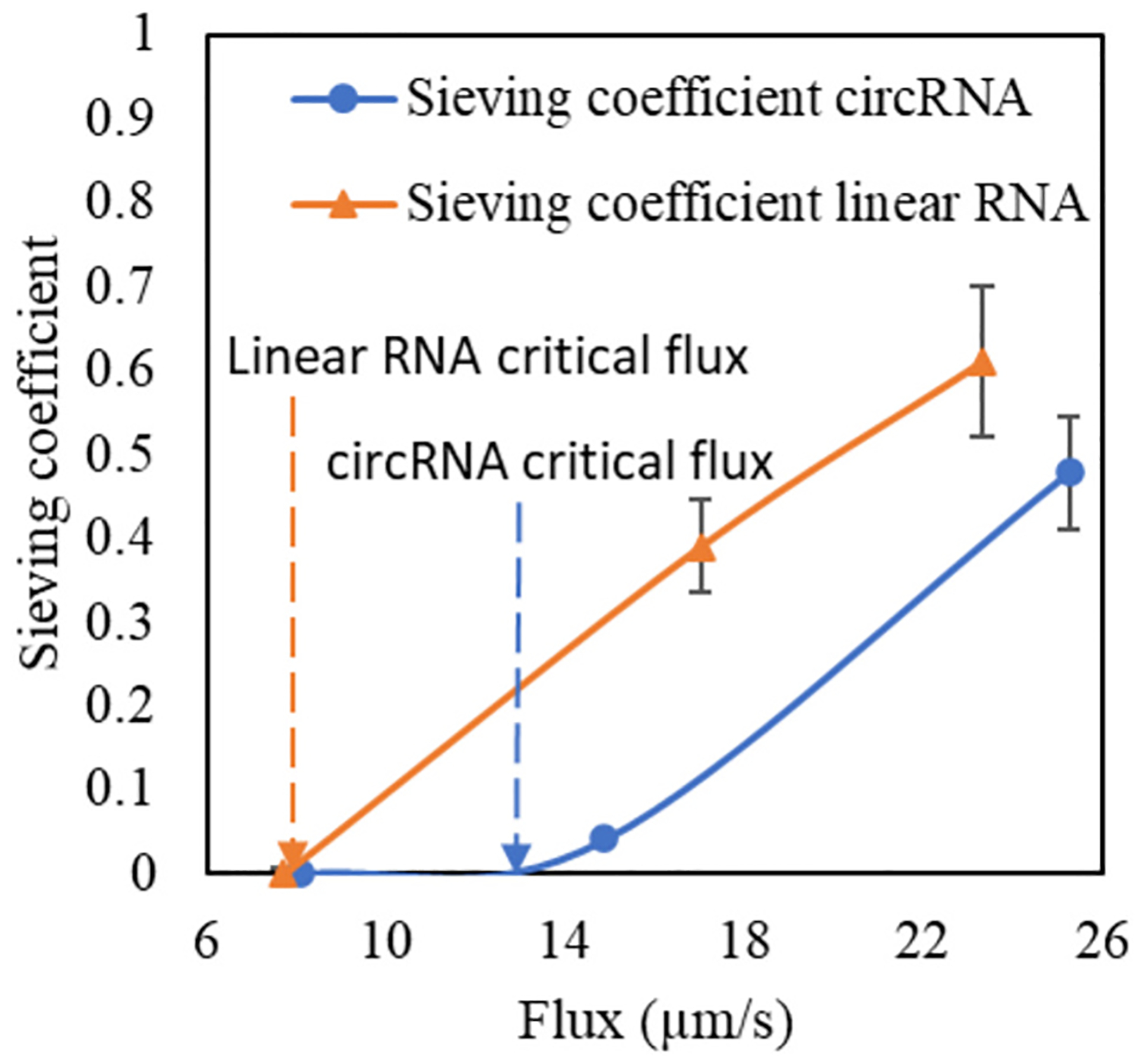
Effect of flux on the sieving coefficients of circular and linear RNA conformers in the IVT reaction mixture for a pore size of 27 nm. The flux between the circular and linear critical flux is the operation window expected to provide high purity and yield.

**Fig. 4.**
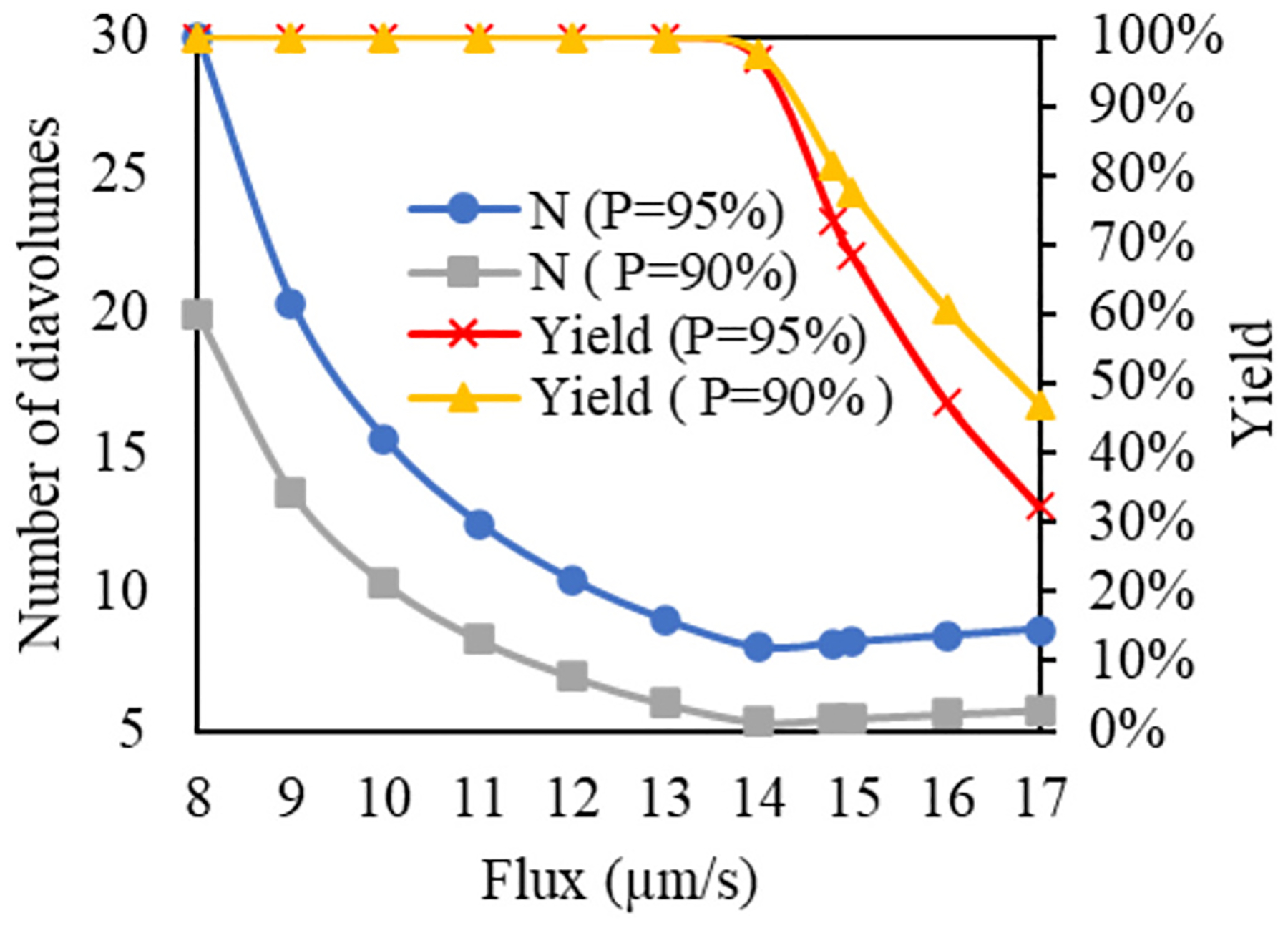
Effect of flux on the number of diavolumes needed to reach a purity between 90 and 95% and the yield we would obtain in each case, according to Eqs. (3) and (4). If the flux is <11 μm/s, the number of diavolumes required to reach 95% is >10, making it impractical. When the flux >15 μm/s, the yield is <70%, which is undesirable because we aim to recover most of the circRNA. A working range between 11 and 15 μm/s balances yield and number of diavolumes. The sieving coefficients used in these calculations were obtained by linear interpolation of the experimental data in Fig. 3 and are intended to provide approximate guidance rather than precise predictions.

Fig. 3 shows the effect of flux on the sieving coefficients of RNA conformers recovered and purified from the IVT reaction mixture. As flux decreases, the sieving coefficient also decreases, reaching zero at the critical flux: 7.7 μm/s for linear RNA, which are the bands around 2 kb in the electrophoresis gel, and 13.9 μm/s for circRNA, which are the two bands around 3 kb. Operating at fluxes between 7.7 and 13.9 μm/s should, in theory, result in 100% purity and 100% yield with sufficient diavolumes, since no circRNA passes through the membrane. However, operating too close to the lower limit (7.7 μm/s) results in low linear RNA transmission, requiring processing many diavolumes to reach our target purity of 95%. For example, using Eqs. (3) and (4), achieving a purity of between 90 and 95% at a flux of 8 μm/s would require from 20 to 30 diavolumes (Fig. 4), which is not feasible in a manufacturing setting, as it would lead to excessive buffer consumption and is therefore unsustainable at scale.

### 3.5. Continuous diafiltration of IVT reaction mixtures

**Fig. 5.**
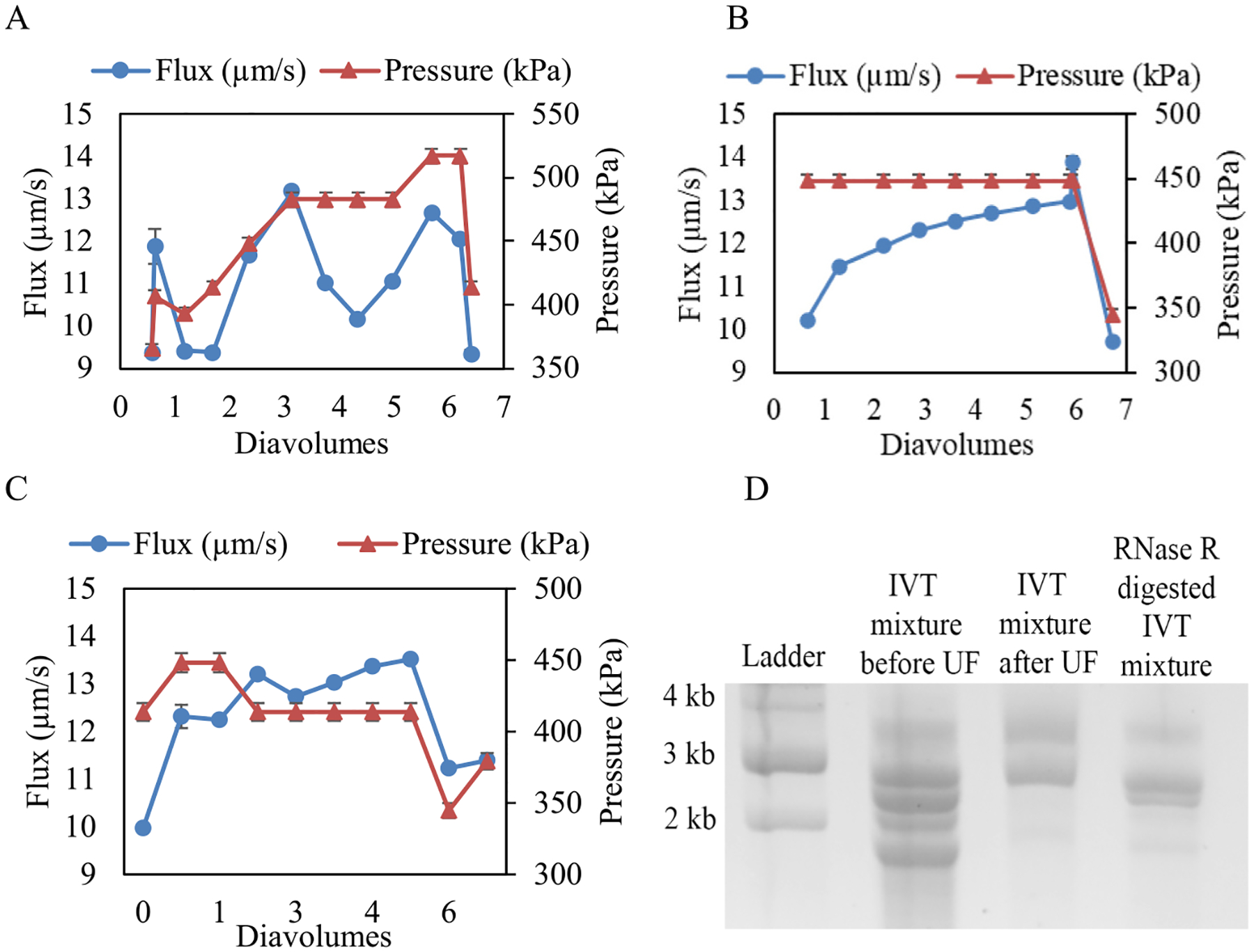
Continuous diafiltration runs and analysis of retained RNA. A) First run using a membrane with a 25 ± 0.8 nm pore diameter and a hydraulic permeability of 0.051 ± 0.001 μm/s/kPa. B) Second run using a membrane with a 24 ± 0.8 nm pore diameter and a hydraulic permeability of 0.046 ± 0.001 μm/s/kPa. C) Third run using a membrane with a 25 ± 0.8 nm pore diameter and a hydraulic permeability of 0.052 ± 0.001 μm/s/kPa. The target flux range of 11–15 μm/s is associated with high-purity, high-yield separation. D) Gel electrophoresis comparison of IVT RNA before and after diafiltration. CircRNA largely remains in the retentate, while linear RNA flows through the membrane. An RNase R-digested circRNA sample was included to confirm the bands corresponding to circRNA.

During continuous diafiltration, the target flux was between 11 and 15 μm/s, as highlighted by shading in Fig. 5. This range was selected because it provides high purity and yield, based on the sieving coefficient analysis. The initial operating pressure was selected from flux-pressure data obtained during hydraulic permeability measurements, with the expectation that flux would decrease when switching from DEPC-treated water to the IVT reaction mixture in TE buffer due to concentration polarization and potential fouling. Pressure was adjusted manually every 30 min to maintain flux within the target range.

Accurate discrimination between circular and linear RNA species is important for quantifying purification performance. In this study, species assignment was based on a combination of RNase R digestion and gel electrophoresis across all diafiltration experiments, with RT-qPCR using junction-spanning primers employed for selected runs to selectively quantify circRNA. While these methods do not provide absolute structural identification of all RNA species present, they were applied consistently within each experiment. Consequently, the reported purity and yield values are interpreted in a comparative manner.

In the initial run (Fig. 5A), maintaining the flux within the desired range proved challenging. Transmembrane pressure was increased gradually during the run to stay within the target flux range. Flux rose in response to each pressure increase but exhibited recurring dips, likely due to concentration polarization and membrane fouling. It never fully stabilized over the course of the experiment. After processing six diavolumes, the pressure was reduced to concentrate the circRNA to <10 mL. In the second run (Fig. 5B), we started with a higher pressure, and the flux was more stable and increased gradually as the number of diavolumes increased, likely due to sample dilution resulting from the addition of pure buffer. Pressure was held constant until six diavolumes were processed. The highest flux was reached at approximately six diavolumes, after which the pressure was reduced to concentrate the circRNA to <10 mL. In the third run (Fig. 5C), pressure was first increased and then decreased as the flux approached the upper limit. It was then held constant, and the flux increased slightly until six diavolumes were processed, at which point we decreased the pressure to concentrate circRNA to <10 mL.

Diafiltered RNA samples collected from these experiments were concentrated by ethanol precipitation. Total RNA concentration was determined by fluorimetry, and composition was analyzed by gel electrophoresis (Fig. 5D). Fig. 5D was the result of the first continuous diafiltration experiment, described in Fig. 5A. RNase R-digested circRNA was run alongside diafiltrated products to compare band patterns. We visually observed high purity in both samples; however, purity could not be quantified from the gel because only the circRNA bands were visible. Purity was therefore determined from the concentration difference between RNase R-digested and undigested samples. Using Eqs. (5) and (6), we calculated a purity of 94% and a yield of 59%.


(5) $$\text{Purity}=\frac{\text{Mass}\;\text{of}\;\text{circRNA}\;\text{in}\;\text{the}\;\text{retentate}\;}{\text{Total}\;\text{mass}\;\text{of}\;\text{RNA}\;\text{in}\;\text{the}\;\text{retentate}}\times 100\%$$

(6) $$\text{Yield}=\frac{\text{Mass}\;\text{of}\;\text{circRNA}\;\text{in}\;\text{the}\;\text{retentate}\;}{\text{Mass}\;\text{of}\;\text{circRNA}\;\text{in}\;\text{the}\;\text{feed}}\times 100\%$$

The yield is below what we expected from the purified fraction results in Fig. 3, but above what has been obtained using other methods. The lower yield may be due to degradation during diafiltration, as the continuous diafiltration lasted approximately 7 h. The contribution of degradation to yield loss was not explicitly isolated by a control experiment in which RNA was held under identical conditions in the ultrafiltration cell without diafiltration. However, prior studies in our laboratory have shown measurable degradation of IVT RNA on time-scales as short as 2 h, even when samples were maintained in an aluminum bead bath nominally set to 4 °C, during which the sample temperature increased to approximately 10 °C. These observations are consistent with multiple reports documenting the strong temperature dependence of mRNA stability [9,10]. Given the extended duration of the present experiments (~7 h), degradation is therefore expected to contribute to yield loss, although RNA deposition on and within the membrane may also play a role. The purities of the second and third diafiltration runs were determined using RT-qPCR, yielding purities of 95% and 92%, respectively, with yields equivalent to that of the first run. The diafiltered fractions were subsequently combined and used for cell transfection. Compared with our previously reported PES membrane results (86% purity and 54% yield) [5], our new method achieved higher purity (92–95%) and a modest increase in yield (59%). These results demonstrate that modifying the PCTE membrane pore size to approximately 25 nm, achieved through gold plating, enables high-purity and high-yield purification of circRNA.


(3) $$P=\frac{C_{\text{circ},0}e^{-S_{\text{circ}}N}}{C_{\text{circ},0}e^{-S_{\text{circ}}N}+C_{\text{linear},0}e^{-S_{\text{linear}}N}}\times 100\%$$

(4) $$Y=e^{-S_{\text{circ}}N}\times 100\%$$

### 3.6. Transfection efficiency into HEK293T cells

Fluorescence microscope images were used to select the mass of circRNA and harvest time for achieving high transfection efficiency. Using the results summarized in Table 2, we selected a 50 ng mass of circRNA and a 24-h harvest time. This mass was increased from 50 to 500 ng when changing from a 96-well plate to a 12-well plate.

**Fig. 6.**
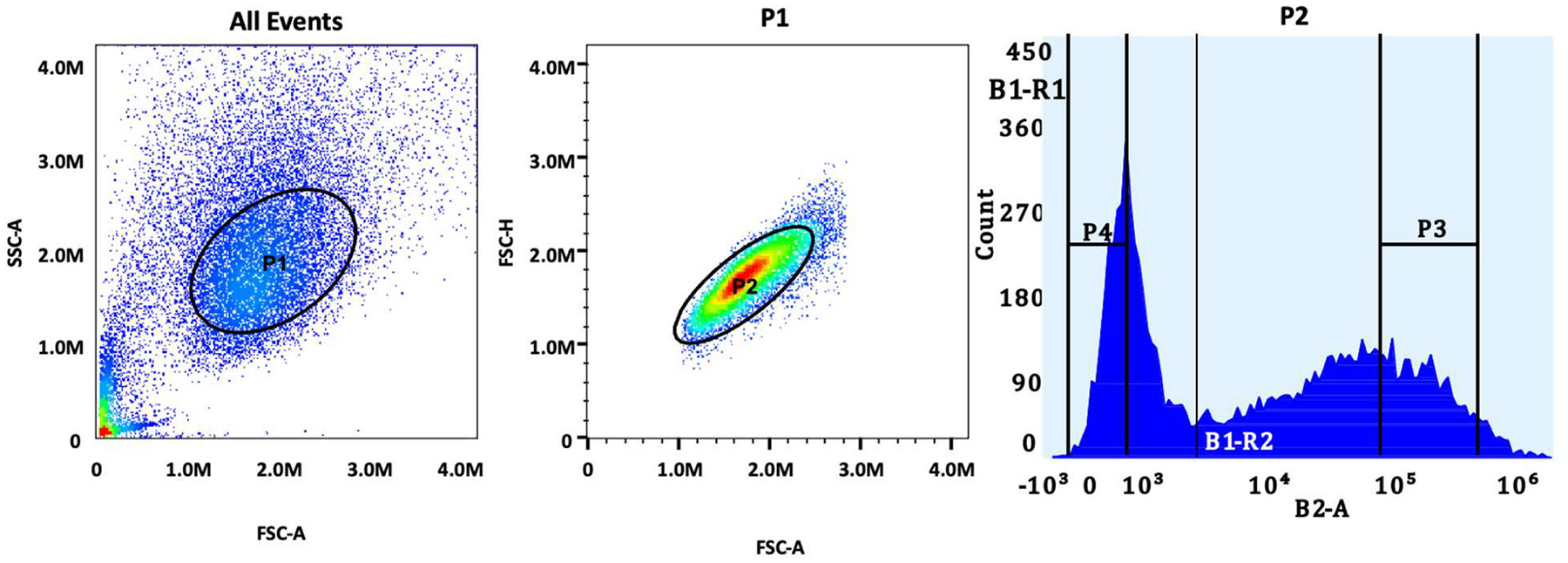
Flow cytometry analysis of HEK293T cell transfection with circRNA. Shown are the gating strategy, fluorescence spectrum, and calculated transfection efficiencies. The B1–R2 gate identifies fluorescent cells within the parent population P2, and the %Parent value (representing the percentage of cells in B1–R2 relative to P2) indicates the transfection efficiency.

Transfection efficiency was determined by flow cytometry, measuring fluorescence in 10,000 individual HEK293T cells. Using SpectroFlo flow cytometry software, total cell counts and fluorescent cell counts were obtained by selecting singlets through appropriate gating (Fig. 6). The transfection efficiency was calculated as the ratio of fluorescent cells to the total cells.

Table 3 summarizes the results. Efficiencies were 28.2% for diafiltrated circRNA and 32.4% for RNase R-digested circRNA starting from a 90% confluency. Markedly higher transfection efficiencies were obtained starting with a lower confluency before transfection. The transfection efficiency of diafiltered circRNA was lower in both cases, indicating that the diafiltration process may influence transfection outcomes. This effect could be associated with residual ethanol from the ethanol precipitation step used to concentrate circRNA after diafiltration.

## 4. Conclusions

This study demonstrated that polycarbonate track-etched (PCTE) membranes with precisely controlled pore sizes can selectively purify circular RNA (circRNA). By applying design equations for continuous diafiltration, we identified operating conditions that achieved 94% purity and 59% yield with only a few diavolumes. These performance metrics are interpreted comparatively, based on consistent analytical methods and orthogonal validation, rather than absolute structural assignment of all RNA species. Despite the effective separation, the purified circRNA exhibited modestly reduced gene expression in HEK293T cells compared with RNase R-treated material.

Several avenues exist to increase yield and product quality. Increasing membrane porosity while retaining uniform pore size would shorten processing times and reduce circRNA degradation. Higher RNA feed concentrations would limit dilution requirements and provide a circRNA product that is immediately suitable for transfection. Additionally, implementing tangential flow filtration, which has demonstrated strong performance for mRNA purification [11,12], may yield similar benefits for circRNA.

Overall, these results establish ultrafiltration with precise pore size membranes as a practical and scalable alternative to chromatographic purification for circRNA. The identified opportunities for process improvements highlight a clear path toward efficient manufacturing of high-quality circRNA therapeutics.

## Figures and Tables

**Table 1:** Effects of gold plating concentration and plating time on hydraulic permeability and estimated pore diameter of PCTE membranes. Hydraulic permeability was measured using flux experiments at multiple transmembrane pressures with water, and pore diameters were estimated from the change in volumetric flow rate.

	Gold plating solution volume (mL)	Plating time (min)	Hydraulic permeability (μm/s/kPa)	Estimated pore diameter (nm)
Batch 1	0.25	45	0.062 ± 0.001	26 ± 0.8
Batch 2	0.50	45	0.035 ± 0.001	23 ± 0.8
Batch 3	0.25	60	0.051 ± 0.002	25 ± 1.0
Batch 4	0.50	60	0.025 ± 0.001	21 ± 0.8
Batch 5	0.25	50	0.067 ± 0.001	27 ± 0.8

**Table 2:** Transfection efficiency for RNase R-digested circRNA at different circRNA concentrations and harvest time.

RNase R-digested circRNA (ng)	Harvest time (h)	Transfection efficiency
30	24	24%
30	17	27%
30	6	15%
50	24	56%
50	17	43%
50	5	11%

**Table 3:** Comparison of the transfection efficiency of diafiltered circRNA and RNase R-digested circRNA.

Confluency	Harvest time (h)	Diafiltered circRNA transfection efficiency	RNase R-digested circRNA transfection efficiency
90	24	28.2%	30.4%
50	24	77.6%	90.6%

## Data Availability

We have shared the link to our data in the Attach file step. Purifying-circRNA-by-ultrafiltration-with-membranes-having-well-defined-pores (Reference data) (GitHub)
